# Emotion Regulation and Eating Disorders in Sports: A Systematic Review

**DOI:** 10.3390/healthcare14060719

**Published:** 2026-03-11

**Authors:** Silvia P. Espinoza-Barrón, Abril Cantú-Berrueto, María Á. Castejón, Rosendo Berengüí

**Affiliations:** 1 Faculty of Sport Organization, Universidad Autónoma de Nuevo León, San Nicolás de los Garza 66455, Mexico; silvia.espinozaba@uanl.edu.mx (S.P.E.-B.); abril.cantubrr@uanl.edu.mx (A.C.-B.); 2 Faculty of Education, Universidad Católica de Murcia (UCAM), 30107 Murcia, Spain; macastejon@ucam.edu

**Keywords:** emotion regulation, eating disorders, cognitive reappraisal, expressive suppression, mental health, risk behavior, prevention

## Abstract

**Background:** Emotion regulation refers to the processes through which individuals influence their emotional experiences, including how emotions are generated, experienced, and expressed. Difficulties in emotion regulation have been identified as a relevant factor in the development and maintenance of Eating Disorders (EDs). In the sports context, high physical and performance demands may intensify emotional challenges, potentially increasing vulnerability to eating disorder symptomatology among athletes. **Objectives**: This systematic review aimed to examine the relationship between emotion regulation and EDs in athletic populations, with a particular focus on emotion regulation strategies and related emotional processes. **Methods**: The PICO model was used, and PRISMA guidelines were followed. The Redalyc, Dialnet, SpringerLink, and PubMed databases were searched from inception to April 2025, with an update in November 2025. After the selection process, nine studies involving athletes from different disciplines and competitive levels were included. Methodological quality and risk of bias were assessed using the Joanna Briggs Institute (JBI) Critical Appraisal Checklists. **Results**: The findings indicate that adaptive emotion regulation strategies, such as Cognitive Reappraisal and emotional identification, are associated with lower levels of eating disorder symptomatology, body dissatisfaction, and greater resilience to sport-related pressures. In contrast, dysfunctional strategies, including expressive suppression, emotional unawareness, and difficulties in emotion management, were consistently associated with restrictive eating behaviors, bulimic symptomatology, excessive weight control, and increased ED risk. Additional emotional factors, including anxiety, perfectionism, low self-esteem, and body image dissatisfaction, were also related to higher vulnerability to EDs, particularly in sports with high aesthetic or weight-related demands. **Conclusions**: Emotional regulation is closely associated with ED risk in athletes. Adaptive emotion regulation strategies may serve as protective factors, whereas dysfunctional strategies are associated with increased risk.

## 1. Introduction

Emotions have been a topic of study in psychology due to their impact on thinking, behavior, and mental health. Traditionally, emotions have been described as arising when an individual evaluates a particular situation and assigns it a meaning related to their goals and concerns. As emotions develop, the individual generates psychophysiological responses that can be perceived as pleasant or unpleasant, depending mainly on their nature, intensity, and duration [1].

In contrast, contemporary models conceptualize emotions as dynamic and context-dependent processes that are actively constructed by the brain. According to this perspective, emotions emerge from ongoing predictive processes that integrate contextual cues, prior experience, and bodily states, with the primary function of supporting physiological regulation and adaptive behavior rather than merely reacting to external stimuli [2,3].

Within these contemporary perspectives, emotions are understood as emerging from regulatory processes and are considered goal-directed processes that support physiological balance and adaptive behavior. From a psychological and applied standpoint, this conceptualization has motivated increasing interest in how individuals can modulate these processes, giving rise to constructs such as emotion regulation. This is particularly relevant in the context of cognitive-behavioral psychology and is defined as a complex process that involves a person’s ability to influence their own emotions, both at the moment they arise and, in the way, they are experienced and expressed [4,5,6].

This process encompasses multiple aspects, as exemplified by the modulation of the physiological response, the implementation of adaptive strategies, and the ability to extrapolate individual strategies to the social context. Despite the many forms that emotion regulation can take, there are three important common factors for adaptive regulation: awareness, goals, and strategies [1].

The Model of Emotion Regulation Process of Gross [5] suggests that people modulate their emotions through strategies that allow them to adapt to the demands of the environment. The model includes five sets of strategies classified as antecedent-focused (what is done before the emotion is totally activated) and response-focused (what is done once the emotion is already being experienced).

From this model, two strategies that have received special attention emerge: Cognitive Reappraisal (CR) and Expressive Suppression (ES). CR is a cognitive strategy focused on antecedents that involves changing the way a situation is interpreted to alter its emotional impact. For example, instead of thinking “I won’t make it,” the person might think “I’m doing the best I can”. ES, on the other hand, is a response-focused strategy that involves hiding, inhibiting, or reducing emotional expressive behavior. For example, a person who hides their frustration might display a polite smile, even though they are feeling that things are not going as expected [7].

Research has shown that CR and ES have different consequences. CR is associated with positive effects, including greater well-being and lower negative affect, while ES is related to negative effects, such as greater negative affect and symptoms related to anxiety or depression [8,9]. It is important to emphasize the connection between emotion regulation and various socio-emotional aspects. In several studies, better regulation is associated with better social adjustment and greater social competence (e.g., emotional knowledge and prosocial response). It is also associated with fewer behavioral problems, even from an early age [10,11]. In adolescents, CR mediates the relation between socio-emotional competencies and anxiety [12]. Furthermore, emotion regulation is associated with better interpersonal skills, in particular empathy, social competence, and relationship quality [13,14,15]. Also, CR is associated with greater cognitive and affective empathy and compassion, and with less empathic distress [16]. Moreover, evidence indicates a close connection between emotion regulation and emotional intelligence (EI). Thus, for example, people with high EI use more adaptive regulatory strategies (as an example, CR, problem-solving, or seeking support) and less avoidance, rumination, or denial [17,18,19], minor ES [19,20], and are more flexible in switching strategies depending on the situation in daily life [21,22].

Contemporary approaches emphasize that emotion regulation does not operate in isolation, but rather as part of a broader network of emotional, cognitive, and contextual processes [2,3]. In applied settings, specifically sport, emotion regulation strategies interact with variables including emotional awareness, emotional recognition, stress reactivity, self-esteem, anxiety, and perceived social and performance-related pressure. These factors may influence both the selection and effectiveness of regulatory strategies and are particularly salient in environments characterized by high physical and psychological demands. Consequently, empirical studies examining emotional processes related to Eating Disorders (EDs) in athletes may operationalize emotion regulation through diverse, but conceptually related, constructs rather than through specific strategies alone. In this context, constructs such as emotional identification (the ability to recognize and label one’s emotional states), emotional unawareness (limited awareness or understanding of emotional experiences), and difficulties in emotion management (problems modulating or responding adaptively to emotional experiences) are frequently examined as indicators of broader emotion regulation capacities.

Recent theoretical and empirical work suggests that the effectiveness of emotion regulation strategies is closely related to interoception, defined as the perception and interpretation of internal bodily signals. Functional regulation strategies, in particular CR, appear to be supported by adequate interoceptive awareness, facilitating the integration of bodily states into adaptive emotional responses. In contrast, maladaptive strategies, such as ES, have been associated with diminished interoceptive processing and reduced sensitivity to internal cues. In the sports context, physical activity has been shown to modulate interoceptive processes, highlighting the relevance of bodily awareness and self-regulatory mechanisms for emotional functioning in athletes. This emerging framework provides a contemporary theoretical background for understanding emotion regulation within physically demanding environments [23,24].

The absence of adequate emotion regulation capacities has been linked to a variety of mental disorders, including Eating Disorders (EDs) [1,25]. EDs are characterized by distorted eating behavior, an extreme concern for self-image and body weight, leading to weight control behaviors (as an example, vigorous physical activity, dietary restriction, fasting, and laxative abuse), resulting in significant deterioration of the individual’s physical, psychological, and social health [26,27,28].

EDs have been associated with difficulties in emotion regulation processes, and it is estimated that between 40% and 75% of people with EDs experience significant challenges in this area, including difficulty in regulating their emotions, altered perception of behavior, and the use of maladaptive strategies [1,29,30].

This high prevalence of difficulties in emotion regulation in individuals with EDs underscores the importance of its association with the development and persistence of these disorders, especially in contexts such as sports, where the pressure and emotional demands of the environment can be particularly intense [31,32].

In sports, emotion regulations have been linked to EDs in various ways. This regulation was shown to be significant in mitigating risky eating behaviors in athletes, and special strategies for proper emotion management (although not clearly operationalized) showed a protective effect against EDs [33]. Also, emotional recognition may function as a protective factor against the development of EDs in women [34]. In addition, in adolescent athletic populations, recent research has begun to explore the relationship between regulation strategies and several risk indicators associated with EDs, providing evidence of the relevance of assessing variables, in particular CR and ES, in this age group [35].

Understanding how emotion regulation is associated with ED risk in athletes allows for the recognition of risk and protective factors, as well as the development of interventions and prevention programs aimed at athletes, improving their mental health and well-being. To date, and based on available information, no systematic review has specifically synthesized evidence on the relationship between emotion regulation and EDs within the sports context. Previous research has primarily examined emotion regulation and EDs in general populations. This lack of systematic synthesis highlights the need for a review that integrates emotion regulation models within sport-specific contexts. Therefore, the present study aims to investigate the relationship between emotion regulation and EDs in the sports context, to understand how emotion regulation and related emotional processes, including specific strategies, namely CR and ES, are associated with ED risk in athletes, considering the influence of the demands of the sports environment.

## 2. Materials and Methods

### 2.1. Study Registration

This systematic review of published studies was conducted following the PRISMA (Preferred Reporting Items for Systematic Reviews and Meta-Analyses) guidelines [36]. After verifying that no similar reviews existed in the database, the review protocol was submitted to PROSPERO (ID: CRD420251237733).

### 2.2. Eligibility Criteria

Eligible study designs included quantitative (cross-sectional, case–control, cohort), qualitative, and mixed-methods studies that reported original data on emotion regulation and ED outcomes in athlete populations. Experimental intervention studies addressing emotion regulation in athletes were also eligible. Only studies published in English or Spanish were considered. Studies were excluded if they did not assess emotion regulation or closely related emotional processes (e.g., emotional awareness, emotional recognition, or emotion-related coping) in relation to ED outcomes. Grey literature (e.g., theses, book chapters, conference proceedings, and non-peer-reviewed reports) was excluded, as the review focused on peer-reviewed journal articles. The time restriction (2010–2025) was applied to focus on recent studies. For synthesis, eligible studies were grouped according to sample characteristics, methodology (design, instruments, and procedures), and results related to emotion regulation and ED outcomes. The criteria for selecting the articles are described in Table 1.

### 2.3. Search Strategy

For the formulation of the research aim, the PICO Definition Model was considered, establishing the search terms to identify the relationship between emotion regulation and EDs in the sports context.

Participants (P): Athletes from different disciplines and levels of experience.

Intervention (I): Emotion regulation processes and related emotional constructs, including specific functional strategies (e.g., CR) and dysfunctional strategies (e.g., ES).

Comparison (C): Comparison across different emotion regulation profiles, strategies, or emotional functioning patterns in relation to eating disorder outcomes.

Outcomes (O): Development or prevention of EDs (such as anorexia, bulimia, orthorexia, etc.).

The terms and combinations used in the information search are described in Table 2. Equivalent adaptations were applied across databases. In each database, search terms were combined using Boolean operators according to the database-specific syntax. An example of a complete search string used was: (“athletes” OR “sport”) AND (“emotion regulation” OR “cognitive reappraisal” OR “expressive suppression”) AND (“eating disorders” OR “EDs”). The following databases were searched from inception to April 2025: Redalyc, Dialnet, SpringerLink, and PubMed. These databases were selected because they provide complementary coverage of biomedical, psychological, and sport-related literature relevant to the topic. The initial search was conducted between February and April 2025. An updated search was performed in November 2025 to identify newly published studies, and eligible records retrieved in this update were included in the review. Reference lists of included studies and relevant reviews were manually screened between May and June 2025. The complete search strategies executed in each database, as an example, exact Boolean strings, are provided in Appendix A.

### 2.4. Selection of Publications

Titles and abstracts were screened by a single reviewer using the predefined inclusion and exclusion criteria. Full-text articles were subsequently assessed for eligibility by the same reviewer. Records were managed manually using Zotero v8.0.4 for reference management. Data extraction was performed manually using a predefined data extraction form. A second researcher conducted a post hoc analysis of the screening decisions and extracted data to verify consistency with the predefined criteria. The second reviewer was not blinded to the initial screening decisions. Any discrepancies were discussed and resolved by consensus. Study authors were not contacted for additional information.

**Figure 1:**
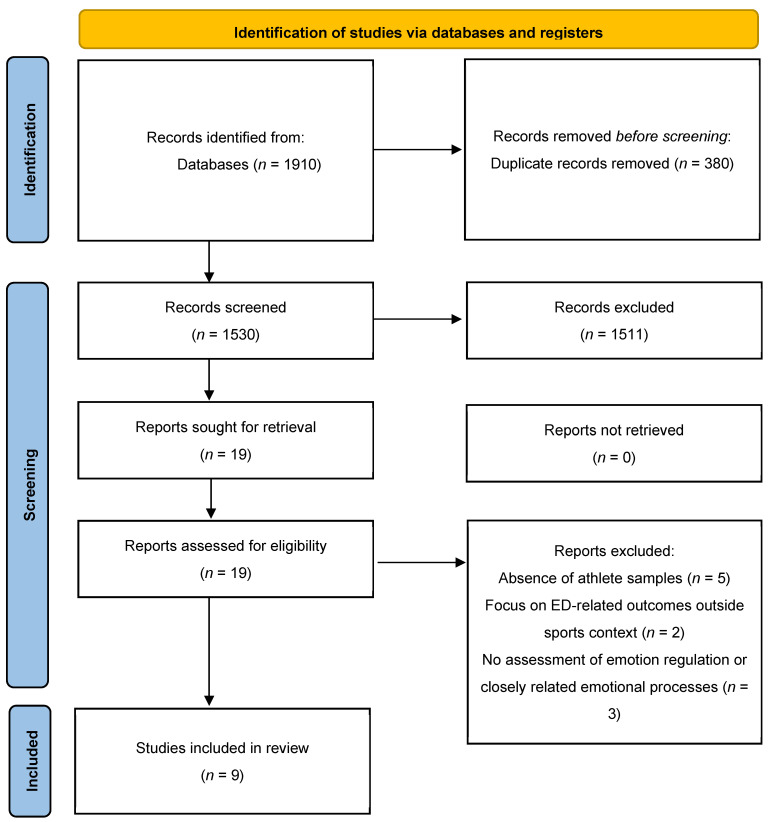
Flow diagram: selection process of studies included in the systematic review.

Following the PRISMA methodology, the initial search in the databases yielded 1910 results, of which duplicate records were identified and removed manually by comparing titles, authors, and journal sources across databases using Microsoft Excel (Figure 1). Subsequently, a complete analysis of the title was conducted, followed by a review of the abstract, and finally, a review of the full text using the inclusion and exclusion criteria. The total number of articles included was 9.

The data were collected in Microsoft Excel, considering the following data blocks: (a) academic data such as year of publication, authors and country of origin, and country of publication; (b) methodological perspective; (c) sample; (d) measurement instruments; and (e) main findings related to the relationship between emotion regulation strategies and eating disorder outcomes in sports, incorporating evidence closely related to emotional factors and eating disorder outcomes in athlete populations. For synthesis, extracted findings were subsequently grouped and analyzed thematically according to the type of emotion regulation process involved (adaptive strategies, maladaptive strategies/dysregulation, and broader emotional or psychosocial vulnerability factors), following a narrative thematic synthesis approach.

Methodological quality and risk of bias of the included studies were assessed post hoc using the Joanna Briggs Institute (JBI) Critical Appraisal Checklists for analytical cross-sectional studies. This assessment was conducted after study inclusion to evaluate the internal validity and methodological quality of the evidence base. Studies were classified as presenting low, moderate, or high risk of bias based on the proportion of criteria met, with ≥75% classified as low risk, 50–74% as moderate risk, and <50% as high risk of bias. Several studies that initially appeared to meet the inclusion criteria were excluded after full-text assessment. The main reasons for exclusion were: (a) absence of athlete samples; (b) focus on EDs related outcomes outside the sports context; and (c) lack of assessment of emotion regulation or closely related emotional processes as defined by the theoretical framework of the present review. Specifically, some studies examined broader psychological constructs without a clear conceptual or operational link to emotion regulation processes relevant to ED outcomes in athletes.

## 3. Results

### 3.1. Risk of Bias

As shown in Table 3, the overall methodological quality of the included studies was acceptable, with most studies presenting either a low or moderate risk of bias. Studies classified as low risk generally demonstrated greater methodological rigor through clearer handling of potential confounding variables, whereas those rated as moderate risk were primarily limited by insufficient identification of confounders and the absence of explicit strategies to control their effects. Importantly, no study was classified as having a high risk of bias, suggesting that the evidence base is reasonably robust. Nevertheless, the presence of unresolved confounding in several studies indicates that findings should be interpreted with caution, particularly when concluding the strength and specificity of associations between emotion regulation and ED outcomes in athletic populations.

### 3.2. Academic Data of the Publication and Methodological Perspective

A total of 9 scientific articles were considered that met the inclusion criteria associated with the relationship between emotion regulation and EDs in the sports context. These articles were published between 2015 and 2025. Within these articles, 33 authors from 5 different countries (Spain, Mexico, Portugal, the United States of America, and Australia) participated. The years with the highest number of publications were 2015 and 2020 (*n* = 2). Regarding the methodological approaches, the studies used quantitative methods (*n* = 8) and mixed methods (*n* = 1).

### 3.3. Samples

The total number of participants was 1445, who were active athletes from various sports disciplines. The sample included 910 women (63%) and 535 men (37%). The age of the participants ranged from 10 to 66 years, with an average age of 21.17 years (SD = 4.69). Among the sports modalities included were classical dance, gymnastics, cycling, soccer, rowing, skating, among others.

### 3.4. Measuring Instruments

Regarding the evaluation methods, the reviewed studies employed a broad range of psychometric instruments and qualitative techniques to examine eating disorder symptomatology, emotion regulation strategies, related emotional processes, and complementary contextual variables within athletic populations.

Eating disorder symptomatology was most frequently assessed using the Eating Disorders Inventory (EDI) and its different versions (EDI, EDI-2, EDI-3, and EDI-3 Referral Form), which appeared as the primary measurement tool across studies (*n* = 4) [34,35,37,38].

Emotion regulation was most measured using instruments derived from Gross’s process model. The Difficulties in Emotion Regulation Scale (DERS) and its abbreviated version (DERS-18) emerged as the most frequently used tools for assessing global emotion dysregulation, including impulse control difficulties, lack of emotional clarity, and limited access to regulation strategies (*n* = 3) [34,39,40].

In addition, strategy-specific assessment of CR and ES was conducted using the Emotion Regulation Questionnaire for Children and Adolescents (ERQ-CA) in adolescent athletic samples (*n* = 1) [35].

Consistent with contemporary conceptualization of emotion regulation as a network of interrelated processes, several studies operationalized emotional functioning through constructs closely linked to regulation capacity. This included alexithymia, assessed with the Toronto Alexithymia Scale (TAS-20) (*n* = 1), emotional recognition evaluated through Reading the Mind in the Eyes Task (RMET) (*n* = 1), and social anxiety, measured with the Social Phobia Inventory (SPIN) (*n* = 1) [34].

Cognitive and self-regulatory processes relevant to body image and emotional control were evaluated using instruments such as the Cognitive Fusion Questionnaire—Body Image (CFQ-BI), the Perfectionistic Self-Presentation Scale—Body Image (PSPS-BI), the Emotional Impulsivity Scale (EIE), and the Multidimensional Inventory of Perfectionism in Sport (MIPS) (see Table 4). Together, these instruments capture mechanisms that influence the selection, implementation, and effectiveness of emotion regulation strategies in high-demand sporting contexts.

Several studies incorporated complementary measures to contextualize emotion regulation and ED risk within the broader psychosocial environment of sport. Global psychological functioning and well-being were assessed using the Ryff Scales of Psychological Well-Being (*n* = 1) [42] while self-evaluative constructs were measured through the Self-Esteem Inventory (SEI) (*n* = 1) [37], and the AF5 Self-Concept Questionnaire (*n* = 1) [38]. Sport-specific contextual pressures and motivational factors were captured using the Weight Pressures in Sport—Females (WPS-F) (*n* = 1) [42] and the Test of Specific Motivation toward Dance (AMPET) (*n* = 1) [37].

Finally, qualitative methodologies were used to complement quantitative assessment in one study, employing focus groups to explore athletes’ subjective experiences (*n* = 1) [43].

### 3.5. Thematic Synthesis of Emotion Regulation and Eating Disorder Risk in Athletes

To address the heterogeneity of designs, samples, and outcome measures, a thematic synthesis was conducted. Given the conceptual overlap between emotion regulation strategies and broader emotional processes across the included studies, the findings were organized into thematic domains to facilitate a more integrative interpretation of the evidence. Specifically, results were structured around three predominant themes: (1) adaptive emotion regulation strategies and protective emotional processes, (2) maladaptive emotion regulation strategies and emotion dysregulation, and (3) broader emotional and psychosocial factors related to ED vulnerability in sport contexts. This approach allows for a comparative interpretation of results across studies and athletic populations.

Regarding adaptive emotion regulation strategies (Table 5), evidence suggests a protective association between adaptive emotional regulation and ED risk in athletes. Specifically, a greater ability to identify emotions was associated with increased resilience to social and sport-related pressures linked to eating disorder vulnerability in women, indicating the protective role of emotional recognition within the sport context [34]. In addition, CR was negatively associated with body dissatisfaction and bulimic symptomatology in adolescent athletes [35]. Beyond specific regulation strategies, evidence from an education-based intervention in adolescent dancers indicates that broader emotional skills may function as protective processes, improving psychosocial functioning without increasing ED risk [37].

In contrast, maladaptive emotion regulation strategies and dysregulation-related processes were consistently associated with increased eating disorder symptomatology across athletic populations and sport modalities (Table 6).

ES emerged as a maladaptive emotion regulation strategy positively associated with drive for thinness, body dissatisfaction, and bulimic symptomatology in adolescent athletes, with higher suppression scores observed among those classified as being at risk for EDs [35]. In team sport contexts, higher levels of global emotion dysregulation were associated with risky eating habits and lower body satisfaction in football players [39]. In endurance sports, emotion dysregulation strengthened the association between shape and weight concerns and muscularity-oriented EDs among cyclists, indicating a moderating role in the expression of eating-related pathology [40].

In addition, cognitive fusion with negative body image thoughts was associated with greater vulnerability to EDs, as athletes who strongly identified with these thoughts tended to adopt perfectionistic body presentation strategies and engage in excessive weight control behaviors [41]. Finally, qualitative evidence highlighted that difficulties in emotional awareness and emotion management (such as anxiety, loss of control, and social comparison) were associated with maladaptive eating-related behaviors, including restrictive eating and concern about body weight, in elite gymnasts [43].

Beyond explicit emotion regulation strategies, several studies highlighted emotion-related vulnerability factors and contextual stressors associated with ED risk in athletic populations (Table 7). Although most studies did not directly assess specific emotion regulation strategies, these emotional and psychosocial factors appear to interact with eating-related vulnerability within the sports environment.

Body image dissatisfaction emerged as a central emotional factor associated with greater ED risk across multiple sport contexts, including dance, gymnastics, cycling, and rowing [37,40,42,43].

Lower emotional self-concept and reduced self-esteem were also linked to higher levels of eating disorder symptomatology in physically active populations, suggesting heightened emotional vulnerability among affected athletes [38].

In team sport settings, athletic identity and perfectionism were identified as relevant psychological vulnerability factors associated with ED risk, particularly among football players [39]. Similarly, perfectionistic tendencies related to body image were associated with EDs in aesthetic sports, highlighting their relevance across different sport modalities [41].

Finally, perceived weight and appearance-related pressure were associated with increased eating-related vulnerability in sports emphasizing body control, to illustrate rowing and aesthetic disciplines [42,43]. Across sport contexts, this vulnerability appeared to be further exacerbated when body-related concerns co-occurred with elevated emotional dysregulation [40].

In summary, the 9 studies reviewed suggest that emotion regulation strategies and emotional factors are relevant correlates of ED risk in athletes, highlighting the importance of emotional functioning within the sporting environment.

## 4. Discussion

This research aimed to investigate the relationship between emotion regulation and EDs in the sports context in order to understand how emotion regulation strategies and related emotional processes are associated with the development and persistence of these disorders. Overall, the findings synthesized across studies indicate that the ED risk in athletes is closely linked to how emotions are regulated within demanding sport environments. Specifically, the evidence converges around three interrelated domains: adaptive emotion regulation strategies that appear to function as protective processes, maladaptive regulation strategies, and broader emotion dysregulation associated with increased vulnerability, and emotional and psychosocial contextual factors that interact with regulation processes to shape ED risk.

First, the thematic synthesis suggests that adaptive emotion regulation strategies play a protective role against eating disorder symptomatology in athletes. Across studies, abilities, among them emotion recognition and CR, were associated with lower body dissatisfaction, fewer bulimic symptoms, and greater resilience to social and sport-related pressures [34,35]. These findings align with Gross’s Model [5], in which strategies implemented earlier in the emotional process allow individuals to reinterpret emotionally salient situations before maladaptive responses are consolidated. Consistently, previous research in the general population has shown that CR is associated with lower eating disorder symptomatology and greater psychological adjustment [44,45].

In the sports context, such strategies may help athletes manage performance-related stressors and body-related concerns more flexibly and adaptively, thereby reducing vulnerability to disordered eating. Furthermore, these strategies have been associated with socioemotional benefits. Emotional regulation, both intrapersonal and interpersonal, has been linked to improvements in performance and the quality of interpersonal relationships [46,47,48]. Likewise, it is associated with better interpersonal functioning, greater collaboration and communication, especially in team sports [49,50,51], which translates into more positive emotional states, increased motivation, and improved collective performance [47]. Furthermore, satisfying the need for relatedness promotes the use of CR, the experience of pleasant emotions, and adaptive and functional states for performance [52]. In young athletes, higher CR and lower ES are associated with greater levels of enjoyment, confidence, and satisfaction, a stronger intention to continue participating in sports, and less social isolation [53]. In conjunction, these findings suggest that emotion regulation can serve a dual protective function, not only reducing the ED risk but also fostering socioemotional resources associated with mental health, well-being, and performance. This preventive perspective aligns with emerging intervention research in adolescents, where strengthening self-regulation capacities has been associated with improvements in eating behaviors and health indicators [54].

In contrast, maladaptive emotion regulation strategies and broader emotion dysregulation processes were consistently associated with increased vulnerability to EDs across athletic populations. ES, difficulties in emotional awareness and emotion management, and cognitive fusion with negative body image-related thoughts were linked to restrictive eating, excessive weight control, bulimic behaviors, and heightened body dissatisfaction [35,39,40,41,43]. These associations were particularly evident in sport contexts characterized by aesthetic demands or strict weight control, where environmental pressures may amplify emotional dysregulation and promote rigid, maladaptive coping patterns. Within Gross’s framework, response-focused strategies such as ES may limit overt emotional expression without reducing underlying emotional arousal, thereby increasing reliance on maladaptive behavioral regulation through eating-related habits.

Beyond specific regulation strategies, the evidence indicates that vulnerability to EDs in athletes is also shaped by broader emotional and self-evaluative factors, including anxiety, perfectionism, low self-esteem, and body image-related cognitive fusion. Studies conducted in demanding sport contexts have shown that these factors contribute to heightened ED risk, particularly among young athletes [39,42], in line with transdiagnostic models that conceptualize emotion regulation difficulties as shared mechanisms across psychological disorders, including EDs [55,56,57].

Taken together, these findings suggest that ED vulnerability in athletes is better understood as part of broader emotion regulation profiles marked by emotional rigidity, limited emotional awareness, and reduced flexibility in responding to sport-related demands. The expression of these profiles appears to vary across sport contexts. In team sports, global difficulties in emotion regulation were associated with risky eating habits and lower body satisfaction [39]. In endurance and aesthetic sports, emotion dysregulation and cognitive fusion amplified the impact of body- and performance-related concerns on eating-related pathology [40,41]. Qualitative evidence further shows that anxiety, loss of control, and social comparison are embedded in athletes’ everyday emotional experiences, reinforcing maladaptive eating behaviors [43]. This indicates that the sport environment actively shapes how emotion-related risk processes operate, rather than acting as a neutral context.

Recent theoretical models help to further explain these patterns by highlighting the role of interoceptive processes in emotion regulation and ED risk in athletes. Interoception refers to how individuals perceive, interpret, and integrate internal bodily signals. Functional regulation strategies, particularly CR, rely on adequate access to bodily sensations, allowing athletes to flexibly reinterpret physiological arousal (e.g., fatigue, hunger, or stress) as part of adaptive emotional experience. In contrast, maladaptive strategies such as ES, avoidance, and difficulties in identifying emotions have been associated with reduced interoceptive awareness, which may foster a disconnection between bodily states and emotional meaning [2,23,24].

In the sports context, where physical activity intensifies interoceptive signals, such a disconnection may increase vulnerability to maladaptive compensatory behaviors, including restrictive eating or compulsive exercise. From a broader theoretical perspective, these behaviors can be understood not only as clinical symptoms but also as maladaptive attempts to regulate or control altered bodily experiences occurring from emotional and physiological dysregulation [58,59,60].

Regarding the evaluation instruments used [61,62,63,64,65,66,67,68,69,70,71,72,73,74,75,76,77], the diversity of scales and methodological approaches employed to assess both emotion regulation and EDs reflects the complexity and multidimensional nature of these constructs in the sports context. Eating disorder symptomatology was most frequently assessed using different versions of the Eating Disorders Inventory (EDI) [34,35,37,38]. Emotion regulation, in turn, was mainly measured through self-report instruments derived from Gross’s process model, as an example the DERS and ERQ-based measures [34,35,39,40]. Complementary approaches in the broader field of eating behavior research have conceptualized self-regulation as a multidimensional construct involving goal-directed strategies and the management of temptation, supported by psychometrically validated instruments in young adult populations [78]. Although the instruments used are widely validated and allow comparison across studies, their predominant reliance on self-report constitutes an important limitation in the sports context. These measures may not adequately capture context-dependent, automatic, and embodied emotional processes, for instance, interoceptive awareness, which are particularly relevant during physical activity [24].

Several limitations of the current review and of the available evidence should be acknowledged. The relatively small number of studies included in this review (n = 9) reflects the limited empirical research specifically examining the relationship between emotion regulation and eating disorder outcomes within athletic populations. On the other hand, the scarcity of qualitative or mixed-method studies limits the understanding of athletes’ subjective experiences of emotional management, especially across different stages of sports development and types of sport. In addition, the absence of longitudinal or experimental designs restricts conclusions regarding the directionality and causal nature of the relationship between emotion regulation and ED risk.

In line with these methodological considerations, the risk of bias assessment further contextualizes the strength of the available evidence. Although most of the included studies were rated as having low to moderate risk of bias, a recurrent methodological limitation was the insufficient identification and control of potential confounding variables. This is particularly relevant given the multifactorial nature of both emotion regulation and eating disorder symptomatology in sport, where variables including age, gender, competitive level, and sport-specific pressures may influence observed associations [79,80]. The limited reporting of strategies to address confounding restricts the strength of causal inferences and may partially account for inconsistencies across studies. Nevertheless, the absence of studies with a high risk of bias supports the overall credibility of the evidence and suggests that the observed associations are robust, although they require confirmation through more rigorous longitudinal and experimental research designs.

Finally, regarding the composition of the samples, a predominantly female (63%) and young (*M* = 21.1 years) population was observed, which aligns with the literature that identifies these groups as especially vulnerable to EDs [81,82,83]. This reinforces the importance of designing interventions with a preventive approach, considering characteristics such as gender, life stage, and sports discipline. At the same time, the underrepresentation of male athletes, older age groups, and certain sport modalities highlights the need for future studies to include more diverse samples to achieve a more comprehensive understanding of this issue.

Altogether, these methodological and sample-related constraints highlight the need for more robust and prospective research designs. Future studies should prioritize longitudinal and intervention-based approaches, include more diverse athletic populations, and reduce exclusive reliance on self-report measures to clarify causal pathways and strengthen the ecological validity of emotion regulation-based prevention programs in sport settings.

## 5. Conclusions

The findings of this systematic review confirm that emotion regulation is closely associated with ED risk in athletes. Functional regulation strategies appear to serve as protective resources, whereas maladaptive strategies and broader emotion dysregulation processes are associated with increased vulnerability. Together, these findings underscore the preventive relevance of strengthening adaptive emotional functioning within sport environments.

From an applied perspective, this preventive potential highlights the importance of incorporating emotion-focused components into prevention and intervention programs in sport. However, the evidence suggests that effective approaches should extend beyond the training of isolated regulation strategies (e.g., CR) to also promote broader social–emotional skills. These include emotional awareness, emotional communication, self-compassion, adaptive responses to performance-related stress, and flexibility in dealing with body-related concerns.

In addition, interventions should explicitly address athletes’ relationship with exercise, helping to differentiate adaptive training engagement from compulsive or compensatory exercise behaviors. Programs that integrate emotion regulation with psychoeducation about bodily signals, recovery, and healthy training practices may be particularly relevant in sport contexts characterized by high performance and aesthetic demands.

Coaches, sport psychologists, and program designers should therefore consider implementing practical prevention frameworks that combine emotion regulation training with social–emotional learning and context-sensitive approaches tailored to sport-specific demands. Such programs may enhance athletes’ capacity to manage emotions, interpersonal pressures, and bodily experiences more healthily and sustainably.

Finally, longitudinal and experimental research is needed to clarify causal pathways between emotion regulation processes and ED development in athletes, and to evaluate the effectiveness of integrated intervention models in real-world sport settings.

## Figures and Tables

**Table 1:** Inclusion and exclusion criteria.

Inclusion	Exclusion
From 2010 to 2025 Text in Spanish or English Journal articles Quantitative, qualitative, or mixed method Applied/reported in athletes	Theses, book chapters, systematic reviews, or other types of publications Duplicate articles Articles with partial or no access

**Table 2:** Search terms.

Search Category	Search Terms
Participants	“sport” or “athletes”
Intervention	“emotion regulation” or “emotional management” or “cognitive reappraisal” or “expressive suppression” or “affect regulation” or “affective regulation”
Comparison	“impact” or “effect” or “relation”
Outcomes	“eating disorders” or “EDs”
Combination	Combination of search terms joined by Boolean operators: “AND” and “OR”

**Table 3:** Risk of bias assessment of the included studies.

JBI Item	Benau et al. [34]	Espinoza-Barrón et al. [35]	Requena-Pérez et al. [37]	Castro López et al. [38]	Aguilar [39]	Mazaraki et al. [40]	Paixão et al. [41]	Larrinaga et al. [42]	Cisternas-Bustos et al. [43]
1	Yes	Yes	Yes	No	Yes	Yes	Yes	Yes	Yes
2	Yes	Yes	Yes	Yes	Yes	Yes	Yes	Yes	Yes
3	Yes	Yes	Yes	Yes	Yes	Yes	Yes	Yes	Yes
4	Yes	Yes	Yes	Yes	Yes	Yes	Yes	Yes	Yes
5	Yes	Unclear	No	No	No	Yes	Yes	Yes	No
6	Yes	No	No	No	No	Yes	Yes	Yes	No
7	Yes	Yes	Yes	Yes	Yes	Yes	Yes	Yes	Yes
8	Yes	Yes	Unclear	Yes	Yes	Yes	Yes	Yes	Yes
RISK OF BIAS	Low	Moderate	Moderate	Moderate	Moderate	Low	Low	Low	Moderate

Note: (1) Were the criteria for inclusion in the sample clearly defined? (2) Were the study subjects and the setting described in detail? (3) Was the exposure measured in a valid and reliable way? (4) Were objective, standard criteria used for measurement of the condition? (5) Were confounding factors identified? (6) Were strategies to deal with confounding factors stated? (7) Were the outcomes measured in a valid and reliable way? (8) Was an appropriate statistical analysis used?

**Table 4:** Comparison of studies.

Author	Type of Study	Main Objectives	Sample	Instruments	Data Analysis	Key Findings
Benau et al. [34]	Cross-sectional, non-experimental, correlational-comparative study	Examine psychosocial risk for disordered eating in undergraduate athletes and non-athletes, focusing on gender differences and the role of social cognition, emotion regulation, emotion recognition, and teammate interdependence.	279 undergraduate students (177 females, 102 males); mean age = 19.3 years (SD = 2.24; range = 18–26). The sample included athletes and non-athletes.	Eating Disorders Inventory–3 (EDI-3: Drive for Thinness, Body Dissatisfaction, Bulimia) Toronto Alexithymia Scale—20 (TAS-20: DIF, DDF) Difficulties in Emotion Regulation Scale (DERS) Social Phobia Inventory (SPIN) Reading the Mind in the Eyes Task—Revised (RMET) Self-reported athletic participation (sport type and teammate interdependence).	χ^2^ tests; Mann–Whitney U test; Pearson correlations; Kendall’s τb; 2 × 3 MANCOVA controlling for BMI; Descriptive Discriminant Analysis (DDA).	Eating Disorder (ED) risk in undergraduate athletes showed clear gender-specific patterns, with women reporting higher drive for thinness and body dissatisfaction and men showing greater emotion dysregulation and bulimic symptoms. Emotion regulation and emotion recognition processes differentiated risk profiles, indicating that athletic participation—rather than team interdependence—is associated with distinct emotion-related pathways to disordered eating in men and women, even at recreational levels.
Espinoza-Barrón et al. [35]	Instrumental psychometric study with a non-experimental, cross-sectional, comparative design.	Examine the reliability and structural validity of the ERQ-CA in young Mexican athletes and analyze the associations between emotion regulation strategies (CR and ES) and ED risk factors.	295 Mexican athletes (129 males, 166 females), mean age = 16.91 (SD = 3.21); 70.1% team sports and 29.9% individual sports; predominantly regional-level competitors.	Emotion Regulation Questionnaire for Children and Adolescents (ERQ-CA: Cognitive Reappraisal, Expressive Suppression) Eating Disorder Inventory—3 Referral Form (EDI-3 RF: Drive for Thinness, Body Dissatisfaction, Bulimia).	Descriptive statistics; reliability analysis (McDonald’s ω); Confirmatory Factor Analysis (CFA); multigroup CFA for measurement invariance (sex and age groups); Pearson correlations; t tests; ANOVA; linear regression analyses.	ES was consistently associated with higher ED risk indicators, including drive for thinness, body dissatisfaction, and bulimic symptoms, whereas CR showed a protective association, particularly with body dissatisfaction. These findings support maladaptive versus adaptive roles of emotion regulation strategies in adolescent athletes and identify adolescence as a sensitive developmental period for ED vulnerability.
Requena-Pérez et al. [37]	Non-experimental correlational study with a quasi-experimental pre–post intervention (action research).	Examine the relationships between body image, self-esteem, motivation, and academic performance in young dance students at a conservatory and implement an intervention program to address identified emotional difficulties and improve academic performance.	75 classical dance students (72 females, 3 males), mean age = 15.0 (SD = 1.80), with a mean of 5 years of dance training; an additional control group of 15 adolescent females with ED aged 13–19 years (M = 15.86, SD = 1.80) was included for comparisons.	Eating Disorders Inventory (EDI: body dissatisfaction, drive for thinness, interoceptive awareness, bulimia, ineffectiveness, fear of maturity, perfectionism, interpersonal distrust) General Motivation Questionnaire; Achievement Motivation in Physical Education Test (AMPET: perceived competence, learning commitment, anxiety) Self-Esteem Inventory (SEI).	Descriptive statistics; Mann–Whitney U test for independent samples; correlation analyses between eating disorder symptoms, anthropometric measures, emotional variables, and academic performance; pre–post comparisons following an intervention program.	Adolescent dancers showed mild body dissatisfaction without clinically significant eating disorder symptomatology, suggesting that dance participation alone is not necessarily associated with increased ED risk. Emotional factors—particularly self-esteem, motivation, and stress-related emotional competencies—were strongly associated with academic performance, and an emotional education intervention improved these outcomes, highlighting emotional skills as protective factors in aesthetic sport contexts.
Castro López et al. [38]	Quantitative, non-experimental, cross-sectional correlational study.	Assess levels of eating disorder symptomatology and self-concept in athletes, examine the relationship between symptoms and self-concept, and analyze their associations with sociodemographic variables.	154 athletes engaged in regular resistance training at gyms in Jaén (Spain) (12 females, 142 males), mean age = 24.97 (SD = 6.90).	Sociodemographic questionnaire Eating Disorders Inventory—2 (EDI-2) Self-Concept Form 5 (AF5: academic/work, social, emotional, family, physical).	Descriptive statistics; Pearson correlation analyses; Student’s t tests; analysis of variance (ANOVA).	High levels of ED risk indicators—especially body dissatisfaction, drive for thinness, perfectionism, and fear of maturity—were strongly associated with low emotional and physical self-concept. Lower emotional self-concept emerged as a key vulnerability factor, supporting the role of emotion-related self-evaluative processes in ED risk among physically active populations.
Aguilar [39]	Cross-sectional, descriptive, comparative, and associative (explanatory) study.	Describe subclinical profiles associated with ED risk based on eating habits, body-related concerns, and psychological factors; estimate the prevalence of ED risk in soccer players; examine gender differences; and analyze relationships among ED risk, psychological factors, and satisfaction indicators.	95 federated soccer players (58 males, 34 females) from 11 teams across different competitive levels in Spain, mean age = 22.9 (SD = 5.7); 26% youth and 74% senior players.	Athlete Eating Habits Questionnaire (CHAD: fear of weight gain, weight- and body-related distress, obsessive food concerns, body satisfaction/self-image) Multidimensional Inventory of Perfectionism in Sport (MIPS: striving for perfection, negative reactions to imperfection) State Impulsivity Scale (EIE: gratification, automatism, attentional) Difficulties in Emotion Regulation Scale (DERS) Athletic Identity Measurement Scale (AIMS: social identity, exclusivity, negative affectivity) Manifest Satisfaction Questionnaire (ad hoc: weight, body image, physical capacity, technical talent, performance).	Descriptive statistics; normality testing; Mann–Whitney U test; Student’s t test; Spearman correlation analyses.	Distinct subclinical profiles of ED risk were identified in football players, primarily characterized by difficulties in emotion regulation, strong athletic identity, and perfectionism. Greater emotion dysregulation was consistently associated with risky eating habits and lower satisfaction with body weight, body image, and physical capacity, highlighting emotional dysregulation as a central vulnerability factor in team sport contexts.
Mazaraki et al. [40]	Cross-sectional, non-experimental, correlational study.	Examine factors associated with muscularity-oriented EDs in cyclists, focusing on the roles of shape and weight concern, drive for leanness, and emotion dysregulation, and test whether emotion dysregulation moderates these associations.	139 Australian cyclists (112 men, 27 women), mean age = 38.66 (SD = 11.87); predominantly competitive riders across world to local levels.	Eating Disorder Examination Questionnaire (EDE-Q: Shape and Weight Concern) Drive for Leanness Scale Difficulties in Emotion Regulation Scale—Short Form (DERS-SF) Eating for Muscularity Scale.	Descriptive statistics; Spearman correlations; hierarchical multiple regression analyses with interaction terms (moderation); bootstrapped confidence intervals (1000 resamples); covariates included gender, age, and BMI; Benjamini–Hochberg correction for multiple comparisons.	Muscularity-oriented EDs among cyclists were positively associated with shape and weight concerns and drive for leanness, with emotion dysregulation significantly intensifying these associations. Cyclists with higher body image concerns and poorer emotion regulation showed the greatest vulnerability, indicating a moderating role of emotion dysregulation in endurance sport-related EDs.
Paixão et al. [41]	Cross-sectional, non-experimental correlational study with mediation (path) analysis.	Examine the association between body image-related cognitive fusion and EDs in girls from aesthetic sports, and test whether body image-related perfectionistic self-presentation mediates this relationship.	142 Portuguese female athletes from aesthetic sports (gymnastics, skating, and dance), mean age = 13.97 (SD = 1.67), all actively training.	Body Mass Index z-scores (WHO standards) Cognitive Fusion Questionnaire—Body Image (CFQ-BI) Perfectionistic Self-Presentation Scale—Body Image (PSPS-BI) Eating Disorder Examination Questionnaire (EDE-Q).	Descriptive statistics; Pearson correlations; path analysis (mediation model) using maximum likelihood estimation; bootstrapped indirect effects (5000 resamples); age and BMI controlled as covariates.	Body image-related cognitive fusion was strongly associated with ED severity, both directly and indirectly through body image-related perfectionistic self-presentation. These findings identify maladaptive emotional–cognitive processes related to body image as central mechanisms underlying ED risk in aesthetic sports during adolescence.
Larrinaga et al. [42]	Cross-sectional observational study with a descriptive, comparative, correlational, and explanatory design.	Examine the prevalence of eating disorder symptomatology and weight-related pressure in female fixed-bench rowers, and analyze their associations with self-concept, psychological well-being, sociodemographic factors, sport experience, performance level, and body composition.	208 adult female fixed-bench rowers from northern Spain, mean age = 23.6 (SD = 6.5), competing at two official competitive levels.	SCOFF Questionnaire (eating disorder symptomatology) Weight Pressures in Sport—Females (WPS-F) Physical Self-Concept Questionnaire—Abbreviated (CAF-A) Ryff Scales of Psychological Well-Being Body mass index and body composition (bioelectrical impedance, subgroup).	Descriptive statistics; Pearson’s chi-square tests with Cramér’s V; Pearson correlations; independent samples t-tests with Cohen’s d effect sizes; multiple linear regression models (stepwise OLS) predicting eating disorder symptomatology and weight-related pressure; multicollinearity checks and k-fold cross-validation.	A high prevalence of eating disorder symptomatology was observed in female fixed-bench rowers and was strongly associated with weight-related pressure from teammates and uniforms, as well as a higher self-concept of strength. In contrast, greater psychological well-being, particularly autonomy, and a positive self-concept of physical attractiveness acted as protective factors, underscoring the relevance of psychosocial and emotional processes over objective body composition in ED risk.
Cisternas-Bustos et al. [43]	Non-experimental, cross-sectional, descriptive–correlational study with a mixed-methods approach (quantitative and qualitative).	Analyze risk behaviors and the level of concern regarding body image perception in elite Chilean female gymnasts.	53 elite Chilean female gymnasts, mean age = 15.2 (SD = 2.35), corresponding to 50% of the total population of elite gymnasts in Chile.	Eating Attitudes Test–26 (EAT-26: Dieting, Food Preoccupation, Oral Control) Body Shape Questionnaire (BSQ: body shape concern and body dissatisfaction) Focus groups (qualitative assessment of body image concerns and eating-related risk behaviors).	Kolmogorov–Smirnov test for normality; descriptive statistics (frequencies); χ^2^ (Chi-square) tests for associations; qualitative open, axial, and selective/theoretical coding.	Risky eating behaviors in gymnasts were closely associated with body image dissatisfaction and negative body image perception, despite the absence of clinical ED diagnoses. Sport-specific aesthetic demands and social pressures from coaches, peers, and family reinforced restrictive eating practices, highlighting body image-related emotional and social processes as key vulnerability factors in aesthetic sports.

**Table 5:** Adaptive emotion regulation strategies and protective emotional processes associated with ED risk in athletes.

Emotional Process/Strategy	Association with ED Risk	ED-Related Outcome	Sport/Population Context	Supporting Studies
Emotion recognition	Protective	Greater resilience to sport- and social-related pressures associated with ED risk	Undergraduate athletes	Benau et al. [34]
Cognitive reappraisal	Protective	Lower body dissatisfaction; fewer bulimic symptoms	Adolescent athletes	Espinoza-Barrón et al. [35]
Emotional skills (education-based)	Protective	Improved self-esteem and motivation; no increase in ED risk	Adolescent dancers	Requena-Pérez et al. [37]

**Table 6:** Maladaptive emotion regulation strategies and dysregulation-related processes associated with increased ED risk in athletes.

Dysregulation-Related Process/Strategy	Association with ED Risk	ED-Related Outcome	Sport/Population Context	Supporting Studies
Expressive suppression	Positive association	Drive for thinness; body dissatisfaction; bulimia	Adolescent athletes	Espinoza-Barrón et al. [35]
Emotion dysregulation (global)	Positive association	Risky eating habits; low body satisfaction	Football players	Aguilar [39]
Emotion dysregulation (moderator)	Moderating association	Muscularity-oriented disordered eating	Cyclists	Mazaraki et al. [40]
Cognitive fusion (body image)	Positive association	Restriction; excessive weight control	Aesthetic sports	Paixão et al. [41]
Emotional unawareness/poor emotion management	Positive association (qualitative)	Restrictive eating; anxiety-driven behaviors; social comparison	Elite gymnasts	Cisternas-Bustos et al. [43]

**Table 7:** Emotion-related vulnerability factors and contextual stressors associated with ED risk in athletic populations.

Emotional/Contextual Factor	Association with ED Risk	Sport/Population Context	Supporting Studies
Body image dissatisfaction	Positive association	Dance, gymnastics, cycling, rowing	Requena-Pérez et al. [37]; Mazaraki et al. [40]; Larrinaga et al. [42]; Cisternas-Bustos et al. [43]
Low emotional self-concept	Positive association	Physically active populations	Castro-López et al. [38]
Athletic identity	Positive association	Football players	Aguilar [39]
Perfectionism	Positive association	Football; aesthetic sports	Aguilar [39]; Paixão et al. [41]
Weight- and appearance-related pressure	Positive association	Rowing; aesthetic sports	Larrinaga et al. [42]; Cisternas-Bustos et al. [43]

## Data Availability

No new data were created or analyzed in this study.

## Abbreviations

The following abbreviations are used in this manuscript:

AMPET	Test of Specific Motivation toward Dance
BSQ	Body Shape Questionnaire
CFQ-BI	Cognitive Fusion Questionnaire–Body Image
CR	Cognitive Reappraisal
EAT	Eating Attitudes Test-26
ES	Expressive Suppression
EDI	Eating Disorders Inventory
EDs	Eating Disorders
EIE	Emotional Impulsivity Scale
DERS	Difficulties in Emotion Regulation Scale
ERQ-CA	Emotion Regulation Questionnaire for Children and Adolescents
M	Mean
MIPS	Multidimensional Inventory of Perfectionism in Sport
n	Number of Publications; Number of Studies
PICO	Participants, Intervention, Comparison, Outcomes
PRISMA	Preferred Reporting Items for Systematic Reviews and Meta-Analyses
PROSPERO	International Prospective Register of Systematic Reviews
PSPS-BI	Perfectionistic Self-Presentation Scale–Body Image
SCOFF	SCOFF Questionnaire (Sick, Control, One, Fat, Food)
SD	Standard deviation
SEI	Self-Esteem Inventory
SPIN	Social Phobia Inventory
TAS-20	Toronto Alexithymia Scale
WPS-F	Weight Pressures in Sport-Females

## Appendix A. Database-Specific Search Strategies

**Database**	**Search Strategy**
**Redalyc**	(“regulación emocional” OR “manejo emocional”) AND (“trastornos de conducta alimentaria” OR “trastornos alimentarios”) AND (“deporte” OR “deportistas” OR “atletas”)
	(“reevaluación cognitiva” OR “supresión expresiva”) AND (“trastornos de conducta alimentaria” OR “trastornos alimentarios”) AND (“deporte”)
	(“reevaluación cognitiva” OR “supresión expresiva”) AND (“trastornos de conducta alimentaria” OR “trastornos alimentarios”) AND (“deporte”)
	(“emotion regulation” OR “emotional management”) AND (“eating disorders” OR “eating disorder”) AND (“sport”)
**Dialnet**	“regulación emocional” AND “trastornos alimentarios” AND “deporte”. Note: Due to character limitations imposed by Dialnet, a simplified search string was applied.
**SpringerLink**	(“eating disorder”) AND (“emotional regulation”) AND (“sport”)
	(“emotion regulation” OR “emotional management” OR “cognitive reappraisal” OR “expressive suppression”) AND (“eating disorders” OR “eating disorder”) AND (“sport” OR “athletes”)
**PubMed**	(“emotion regulation” OR “emotional management” OR “cognitive reappraisal” OR “expressive suppression” OR “affect regulation” OR “affective regulation”) AND (“eating disorders” OR “eating disorder” OR “ED”) AND (“sport” OR “athletes”)
	(“Emotional regulation”) AND (“Eating disorders”) AND (“athletes”)
